# Supplementary material for: Identification of photoperiod-induced specific miRNAs in the adrenal glands of Sunite sheep (Ovis aries)
Source: Front Vet Sci. 2022 Jul 22;9:888207. doi: 10.3389/fvets.2022.888207 (PMC9354845; doi:10.3389/fvets.2022.888207)
Supplement: Supplementary file 1 [file Data_Sheet_1.ZIP › Supplementary file/miRDeep2 file.zip!/miRDeep2 file/Know_Structure/SPLP42A1/oar-mir-29b-1.pdf]

miRBase precursor : oar-mir-29b-1
Total read count : 10935
oar-miR-29b read count : 10131
remaining reads : 804

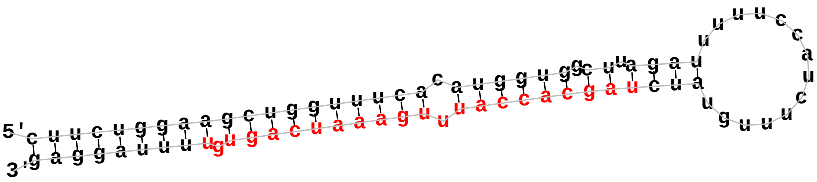

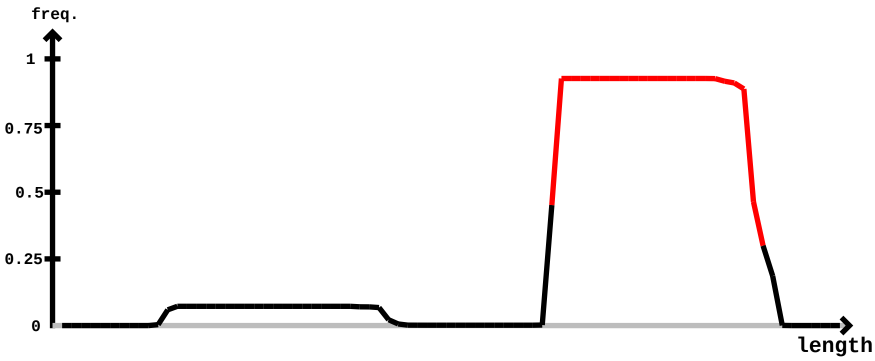

oar-miR-29b

```
5'- cuucuggaagcugguuucacaugguggcuuagauuuuuccaucuuuguaucuagcaccauuugaaaucaguguuuuaggag   -3'    exp
    (((((((((((((((((((.((((((.((.((((..............)))))))))))).)))))))))).)))))))))    reads      mm         sample
    .........gcugguuucacaugguggcu....................................................    1          0          seq
    .........gcugguuucacaugguggcuu...................................................    5          0          seq
    .........gcugguuucacaugguggcuua..................................................    1          0          seq
    .........gcugguuucacaugguggcuuag.................................................    3          0          seq
    .........gcugguuucacaugguggcuuaga................................................    27         0          seq
    .........gcugguuucacaugguggcuuagauuu.............................................    1          0          seq
    ..........cugguuucacaugguggcuu...................................................    13         0          seq
    ..........cugguuucacaugguggcuua..................................................    2          0          seq
    ..........cugguuucacaugguggcuuag.................................................    14         0          seq
    ..........cugguuucacaugguggcuuaga................................................    433        0          seq
    ..........cugguuucacaugguggcuuagau...............................................    114        0          seq
    ..........cugguuucacaugguggcuuagauu..............................................    29         0          seq
    ..........cugguuucacaugguggcuuagauuu.............................................    1          0          seq
    ..........cugguuucacaugguggcuuagauuuu............................................    1          0          seq
    ...........ugguuucacaugguggcuu...................................................    2          0          seq
    ...........ugguuucacaugguggcuua..................................................    1          0          seq
    ...........ugguuucacaugguggcuuag.................................................    10         0          seq
    ...........ugguuucacaugguggcuuaga................................................    53         0          seq
    ...........ugguuucacaugguggcuuagau...............................................    59         0          seq
    ...........ugguuucacaugguggcuuagauu..............................................    20         0          seq
    .................................uuuuucUaucuuuguauc..............................    1          1          seq
    .................................uuuuuccaucuuuguauU..............................    1          1          seq
    .................................uuuuuccaucuuuguauc..............................    10         0          seq
    .................................uuuuuccaucuuuguaucu.............................    1          0          seq
    ..................................uuuuccaucuuuguauU..............................    1          1          seq
    .................................................ucuagcaccauuugaaaucagu..........    8          0          seq
    ..................................................cuagcaccauuugaaau..............    5          0          seq
    ..................................................cuagcaccauuugaaauc.............    49         0          seq
    ..................................................cuaCcaccauuugaaauc.............    2          1          seq
    ..................................................cuagcaccauuugaaauca............    21         0          seq
    ..................................................cuagcaccauuugaaaucG............    2          1          seq
    ..................................................cuagcaccauuugaaaucag...........    43         0          seq
    ..................................................cuagcaccauuugaaaucGg...........    1          1          seq
    ..................................................cuagcaccauuugaaaucGgu..........    19         1          seq
    ..................................................cuagcaccauuugaaaucagu..........    2574       0          seq
```

oar-miR-29b

```
cuucuggaagcugguuucacaugguggcuuagauuuuuccaucuuuguaucuagcaccauuugaaaucaguguuuuaggag
..................................................cuagcaccauuugaaaucagug.........     1341    0    seq
..................................................cuagcaccauuugaaaucagugu........     857     0    seq
..................................................cuagcaccauuugaaaucaguguu.......     5       0    seq
...................................................uagcaccauuugaaauA.............     1       1    seq
...................................................uagcaccauuugaaauc.............     51      0    seq
...................................................uagcaccauuugaaauU.............     4       1    seq
...................................................uagcaccauuugaaauca............     20      0    seq
...................................................uagcaccauuugaaaucG............     28      1    seq
...................................................uagcaccauuugaaaucag...........     55      0    seq
...................................................uagcaccauuugaaaucGg...........     146     1    seq
...................................................uagcaccauuugaaaucagu..........     1444    0    seq
...................................................uagcaccauuugaaaucGgu..........     582     1    seq
...................................................uagcaccauuugaaaucagug.........     460     0    seq
...................................................uagcaccauuugaaaucagugu........     384     0    seq
...................................................uagcaccauuugaaaucaguguu.......     2020    0    seq
...................................................uagcaccauuugaaaucaguguuC......     2       1    seq
...................................................uagcaccauuugaaaucaguguuu......     5       0    seq
....................................................agcaccauuugaaaucagu..........     1       0    seq
....................................................agcaccauuugaaaucagugu........     1       0    seq
```
